# Association of Mediterranean diet, dietary supplements and alcohol consumption with breast density among women in South Germany: a cross-sectional study

**DOI:** 10.1186/1471-2458-13-203

**Published:** 2013-03-07

**Authors:** Olga Voevodina, Christian Billich, Birke Arand, Gabriele Nagel

**Affiliations:** 1 Institute of Epidemiology and Medical Biometry, Ulm University, Helmholtzstr. 22, Ulm 89081, Germany; 2 Departement of Diagnostic and Interventional Radiology, Ulm University, Prittwitzstrasse 43, Ulm, 89075, Germany; 3 Clinic Ludwigsburg, Posilipostr. 4, Ludwigsburg, 71640, Germany

**Keywords:** Mammographic breast density, Diet, Supplements, Alcohol, Breast cancer

## Abstract

**Background:**

Effects of dietary factors, such as adherence to Mediterranean diet, multivitamin-multimineral supplements use and alcohol consumption on mammographic breast density, an important biomarker of breast cancer risk, are not sufficiently consistent to elaborate preventive recommendations. This study aims to investigate the association between current diet and mammographic density.

**Methods:**

We performed a cross-sectional study in 424 pre- and post-menopausal women aged 21 to 84 years. Current Mediterranean dietary pattern, multivitamin-multimineral supplements use, alcohol consumption and potential confounders were assessed with a self-administered questionnaire in the University Hospital Ulm (2007–2008). Radiologists evaluated mammographic density according to the American College of Radiology (ACR) classification, which was summarized in low = ACR1/2 and high = ACR3/4 mammographic density. Logistic regression models were used to assess the association between current diet and mammographic density.

**Results:**

Adherance to Mediterranean dietary pattern was inversely associated with mammographic density in the models adjusted for age and BMI (per 1 unit increase of score OR 0.95; 95%CI 0.90–0.997). Current use of multivitamin-multimineral supplements was also inversely associated with mammographic density (OR 0.53; 95%CI 0.34–0.83). Further adjustment revealed similar point estimates but the associations were no longer statistically significant. Compared to non-drinkers, excessive alcohol consumption (<10 g/d) was positively associated with mammographic density (OR 1.47; 95%CI 0.82-2.63).

**Conclusions:**

Our results show that dietary factors are associated with mammographic density. Adherence to Mediterranean diet and current use of multivitamin-multimineral supplements could be inversely associated with mammographic density and may suggest a protective effect against breast cancer, whereas high alcohol consumption was associated with increased mammographic density.

## Background

Breast cancer is a malignant tumor, originating from the breast’s glandular tissue. It is one of the most frequent malignant tumors in most Western countries [1]. Breast cancer is a common cause of death of women between 40 and 50 years of age [2]. Risk factors for breast cancer are among many others: age, parity, hormone replacement therapy, menopausal status, and family history of breast cancer [3]. Increased mammographic density is an important independent risk factor for breast cancer [4]. High breast density is consistent with the breast’s amount of fibroepithelial tissue [5], which appears as radiographic dense on mammograms [5]. The biological pathways linking high mammographic density with breast cancer still need further investigation.

Mammography is a radiological method for the measurement of breast density [6] and for early detection of breast cancer [7]. Different scales can be used to evaluate the mammographic density. The first classification was established by Wolfe in 1976 [8]. Another classification, BI-RADS with ACR-categories, was developed by the American College of Radiology and published in 1993 [7]. It helped to standardize mammography reports, breast density evaluation and the interpretation of mammograms [7]. Other methods to quantify breast density, like ultrasound and magnetic resonance imaging, also applied in breast diagnostic.

Breast density may be a possible surrogate marker in the prevention of breast cancer [9]. It is important to identify factors which modify mammographic density and thereby might be useful as a basis of preventive interventions. There is evidence that exogenous hormones, a larger number of live births, age, body-mass-index and other factors can alter the pattern of breast density on a mammogram [3]. Especially adherence to Mediterranean dietary pattern, intake of multivitamin-multimineral supplements and alcohol consumption could be established as basic interventions in prevention of high mammographic density in the future [4,9]. These factors show an association with breast cancer risk and more easily modifiable than reproductive factors. Alcohol consumption may have an influence on breast neoplasm formation [10]. It remains unclear whether alcohol consumption increases the mammographic density, compared to non-drinkers. Breast cancer prevalence is lower in Mediterranean populations than in northern European ones [11]. The differences in prevalence could be explained by different dietary patterns in Mediterranean and northern European populations. A Mediterranean dietary pattern is characterized by regular consumption of fruit, vegetables and olive oil [11]. The northern European diet is characterized by high consumption of meat and saturated fatty acids [11]. It may be important to clarify whether an association between the mammographic density of breast tissue and an adherence to Mediterranean diet exists.

This study aims to investigate the association between Mediterranean dietary pattern, current intake of multivitamin-multimineral supplements, and alcohol consumption with mammographic density.

## Methods

### Study population

Between April 2007 and September 2008 494 women aged 21 to 84 years, who underwent mammography at the breast center in University Hospital Ulm, were recruited in this cross-sectional study. After informed consent they completed a self-administered questionnaire. From the statistical analysis 65 participants were excluded due to history of breast ablation and breast cancer and 5 participants filled in the questionnaire twice. Thus 424 women remained for the analysis.

A study protocol was approved by the ethics committee of the medical faculty of Ulm University.

### Data assessment

The questionnaire assessed socio-demographic characteristics, including date of birth, nationality, body weight, height and education. Furthermore, participants were asked to comment on their consumption of food, beverages, physical activity and their smoking status. The food questionnaire of this study was partially adapted from the food questionnaire of the Million Women Study [12]. The choice of the components was based on the hypothesis that a low consumption of “unhealthy” food, such as meat (contains saturated fatty acids) [13] and a high consumption of “healthy” food, such as fruits (contain antioxidants) [14] protect against a high mammographic density as indicator for higher breast cancer risk. Participants provided information on reproductive factors, such as age at menarche, menopausal status and parity, and family history of breast cancer. Data on hormone replacement therapy, biopsy of breast and past medical history was also included.

In this study, we collected information of the supplementary intake of vitamins and minerals both individually and combined. Due to the small numbers on single vitamin and mineral use, we investigated the association between intake of multivitamin-multimineral supplements and mammographic density. The corresponding question in our questionnaire was: “Do you take vitamins, minerals or dietary supplements regularly?”

Data on the consumption of wine, beer and spirits beverages in glasses per week has been collected to assess a drinking of alcoholic beverages. We calculated how much alcohol in g (gram) each woman consumed according to conversions described in other German studies [15,16]. The following conversions were made:

wine: 1 glass = 0.25 liter [15].

beer: 1 glass = 0.3 liter.

spirits beverage: 1 glass = 0.02 liter.

1 liter wine = 100 g alcohol, and

1 liter beer = 40 g alcohol [16].

There were 25 g alcohol in 1 glass wine; 12 g alcohol in 1 glass bier and 6.2 g alcohol in 1 glass spirits beverage [16]. On the basis of this data, we calculated the total alcohol consumption (wine, beer and spirits beverage) per day (d) in gram for each participant. We created quantitative (increase per 10 g/d) and qualitative variables to analyze alcohol consumption. Categorized variable included four classes: > 10.0 g/d total alcohol; 5.0 - 10.0 g/d total alcohol; 0.1 - 5.0 g/d total alcohol and 0 g/d total alcohol.

We selected the dietary components contributing to a score based on other studies of adherence to Mediterranean diet. Based on other studies on the adherence to Mediterranean dietary pattern [11,17] we selected the dietary components in our study. The following foods were included: boiled and raw vegetables, fruit, fish, nuts and olive oil (pro-Mediterranean diet); butter, beef, pork, sausages, ham, hamburger and lemonade/soft drinks (contra-Mediterranean diet). Points were assigned for pro-Mediterranean food: low consumption = 1 point, regular consumption = 2 points, frequent consumption = 3 points. Points for contra-Mediterranean food were reversed: low consumption = 3 points, regular consumption = 2 points, frequent consumption = 1 point. In cases of no consumption or where no information was provided, 0 points were given. In total, a maximum of 29 points could be achieved. High score indicates adherence to Mediterranean diet.

As potential confounders were considered: physical activity at recruitment (yes/no), Body-Mass-Index (BMI) at recruitment (kg/m^2^), ever use of hormone replacement therapy (HRT) (yes/no), menopausal status at recruitment (proxy variable with premenopausal (<50) and postmenopausal (≥50) women), age at recruitment (years), mother with history of breast cancer (yes/no), school education (9 years of school/13 years of school), ever use of oral contraceptives (yes/no), age at menarche (years), smoker at recruitment (yes/no), ever breast feeding (yes/no), number of live births (quantitative variable; 0,1,2,3,4 or 5 live births), age at the first birth (quantitative variable; years), alcohol consumption at recruitment (yes/no) if not exposure variable.

### Mammographic density assessment

Breast density was assessed by two radiologists of the University Hospital Ulm applying the classification of the American College of Radiology (ACR) including four categories: ACR 1 = almost entirely fat, glandular tissue <25%; ACR 2 = scattered fibroglandular densities (ca. 25–50% of the breast); ACR 3 = heterogeneously breast dense (ca. 51–75% of the breast); ACR 4 = extremely dense (>75% of the breast) [7]. For the analyses the ACR categories were combined: category ACR 1/2 (including ACR 1 and ACR 2) means lower density and category ACR 3/4 (including ACR 3 und ACR 4) means higher density of breast. The measurement of the left breast was incorporated into the statistical analysis. Breast densities were assessed by two radiologists separately to determine inter-rater reliability. The differences in assessment of mammographic density between these two raters were calculated with the kappa-value. According to Altman’s interpretation of kappa-values [18], the inter-rater variability assessed by kappa statistics showed moderate agreement (0.48) for ACR classes, but for the categories ACR1/2 and ACR 3/4 good agreement (0.61) was found.

### Statistical analysis

The *X*^2^-test, the *t*-test and the Wilcoxon-test were used to analyze the significance of different frequencies and distribution, respectively. We defined the level of statistical significance as 0.05. We used these tests to investigate the significance of relationships between possible confounders (such as BMI, education, etc.) and either the target variable (mammographic density) or influencing variables (such as current intake of multivitamin-multimineral supplements, current alcohol consumption and Mediterranean dietary pattern). Tests for trend across quartiles were performed by assigning the mean level within specific quartiles of alcohol intake to all individuals in that quartile, and using this as a continuous variable in a linear regression. Logistic regression models were fitted to obtain Odds Ratios (ORs) with 95% confidence intervals (95%CIs) for high mammographic density. Additionally interactions between influencing variables and the target variable were tested. Variables, which were identified as potential confounders because they had a biological plausibility and statistically significant relationships with the target variable and/or influencing variables, were tested with manual step-by-step regression. A variable showed an effect and was identified as a confounder, if it had changed estimator more than 10%. The parsimonious models were adjusted for age and BMI based on results of the manual step-by-step regression and/or the biological plausibility. These models were computed stratified by smoking status in order to account for differential association according to hormonal and oxidative stress, respectively. Also the fully adjusted models were calculated, adjusted for physical activity, BMI, HRT use, menopausal status, age, mother with history of breast cancer, school education, ever use of oral contraceptives, age at menarche, smoker at recruitment, number of live births, alcohol consumption. Statistical analysis was assessed with the Analyst Interface of the SAS (Statistical Analysis System) Version 9.2.

## Results

The study sample is characterized in Table 1. There were 150 premenopausal and 274 postmenopausal women included in the study. Mean age of the study sample was 54 (SD 10.5) years. Most women were German (95%) and had completed at least 9 years of school (71%). More than 50% of the women were normal weight (median = 24.46 kg/m^2^) and most women were physically active (76%). Overall, 80% women had ever taken oral contraceptives and 30% hormone replacement therapy. 80% of the women had ever breastfed.

**Table 1:** Characteristics of study sample

	n*	**Mean**	**SD**	**Min.**	**Max.**	**Median**
Age (at recruitment) (years)	424	53.98	10.50	21.00	84.00	54.00
BMI (at recruitment) (kg/m^2^)	420	25.23	4.86	17.31	65.76	24.46
Age at menarche (years)	407	13.21	1.49	10.00	19.00	13.00
Age at the first birth (years)	343	25.62	5.10	16.00	45.00	25.00
Number of live births	406	1.70	1.05	00.00	5.00	2.00
		**absolute frequency**	**relative frequency**			
		**(n)**	**(%)**			
Physical activity (at recruitment)	424					
yes		324	76			
no		100	24			
Alcohol consumption (at recruitment)	424					
yes		283	67			
no		141	33			
Smoker (at recruitment)	424					
yes		52	12			
no		372	88			
School education	417					
9 years of school		298	71			
13 years of school		119	29			
HRT (ever)	424					
yes		128	30			
no		296	70			
Use of oral contraceptives (ever)	424					
yes		339	80			
no		85	20			
Mother with history of breast cancer	424					
yes		68	16			
no		356	84			
Menopausal status	424					
premenopausal (< 50)		150	35			
postmenopausal (≥ 50)		274	65			
Breast feeding (ever)	339					
yes		272	80			
no		67	20			

Abbreviations: *n*, number of observations; *SD*, standard deviation; *BMI*, body-mass-index; min., minimum; *max*, maximum; *kg*, kilogram; *m*^*2*^, square meter; *HRT*, hormone replacement therapy.

*Numbers may not add up to 424 due to missing values.

There were 35% of the women who consumed multivitamin–multimineral supplements. Alcohol consumption was reported by 67% and smoking by 12%. Most women consumed wine (61%). High scores of Mediterranean diet score (more than 24 points) indicating adherence to Mediterranean diet was observed in 25% of the women. Overall, 56% of the women had high mammographic density (ACR3/4). Among premenopausal women mammographic dense breast parenchyma was more prevalent (73%) than among postmenopausal women (46%).

Adherence to the Mediterranean diet was inversely associated with mammographic density adjusted for age and BMI (per 1 unit increase: OR 0.95; 95%CI 0.90-0.997) (OR 0.97; 95%CI 0.91–1.02) (Table 2) and in the parsimonious model, of which the later reached statistical significance. Stratification by smoking status in this model revealed a statistically significant association between adherence to Mediterranean dietary pattern and mammographic density (OR 0.95; 95%CI 0.90-0.999) among non-smoking women. No association was found in smoking women (OR 0.97; 95%CI 0.80-1.19). This interaction with smoking was not significant (p-value 0.634)

**Table 2:** Association between Mediterranean diet score and high mammographic density (ACR 3/4)

**Mediterranean diet score (increase per increment)**		
	OR*	**95%CI**
Pre- and postmenopausal (n** = 392)	0.97	(0.91 - 1.02)
	OR***	**95%CI**
Pre- and postmenopausal (n** = 420)	0.95	(0.90 - 0.997)
Premenopausal (n = 149)	0.99	(0.89 - 1.10)
Postmenopausal (n = 271)	0.94	(0.89 - 0.99)
Smoker (n = 51)	0.97	(0.80 - 1.19)
Nonsmoker (n = 369)	0.95	(0.90 - 0.999)

Abbreviations: *n*, number of observations; *OR*, Odds Ratio; *95%CI*, 95% confidence interval.

* Fully adjusted models were adjusted for physical activity, BMI, HRT, menopausal status, age, mother with history of breast cancer, school education, ever use of oral contraceptives, age at menarche, smoker at recruitment, number of live births, alcohol consumption.

**Numbers may not add up to 424 due to missing values.

*** Parsimonious models were adjusted for age and BMI at recruitment.

The results of the multivariate analyses for multivitamin-multimineral supplements are displayed in Table 3. The consumption of multivitamin-multimineral supplements was significantly associated with reduced mammographic density in both the fully adjusted model (OR 0.61; 95%CI 0.38-0.97) and also in the parsimonious model. In postmenopausal women the association was significant (OR 0.51; 95%CI 0.30-0.88), but not among premenopausal women (OR 0.58; 95%CI 0.25-1.36). Stratification by smoking status revealed a statistically significant positive association between multivitamin-multimineral supplements and mammographic density among non-smoking women only (OR 0.52; 95%CI 0.33-0.84). However, the interaction between multivitamin-multimineral supplements and smoking status was not significant (p-value = 0.84).

**Table 3:** The association between intake of multivitamin-multimineral supplements and high mammographic density (ACR 3/4)

**Multivitamin-multimineral supplements (current intake)**		
	OR*	**95%CI**
Pre- and postmenopausal (n^**^ = 392)		
no	1 (ref.)	
yes	0.61	(0.38 – 0.97)
	OR***	**95%CI**
Pre- and postmenopausal (n^**^ = 420)		
no	1 (ref.)	
yes	0.53	(0.34 - 0.83)
Premenopausal (n = 149)		
no	1 (ref.)	
yes	0.58	(0.25 - 1.36)
Postmenopausal (n = 271)		
no	1 (ref.)	
yes	0.51	(0.30 - 0.88)

Abbreviations: *n*, number of observations; *OR*, Odds Ratio; *95%CI*, 95% confidence interval.

* Fully adjusted models were adjusted for physical activity, BMI, HRT, menopausal status, age, mother with history of breast cancer, school education, ever use of oral contraceptives, age at menarche, smoker at recruitment, number of live births, alcohol consumption.

**Numbers may not add up to 424 due to missing values.

*** Parsimonious models were adjusted for age and BMI at recruitment.

The results between alcohol consumption and mammographic density are displayed in Table 4. In the parsimonious models, no statistically significant association was found between alcohol consumption and mammographic density in pre- and postmenopausal women (alcohol consumption (>10g/d) versus no consumption (OR 1.47; 95%CI 0.82-2.63). In the fully adjusted model, a significant association emerged (OR 1.94; 95%CI 1.02-3.71).

**Table 4:** Association between alcohol consumption and a high mammographic density (ACR 3/4)

**Alcohol consumption classes (current intake)**		
	OR*	**95%CI**
Pre- and postmenopausal (n** = 392)		
Non-consumer	1 (ref.)	
0.1 - 5.0 g/d	1.77	(0.92 - 3.40)
5.0 - 10.0 g/d	1.85	(0.98 - 3.48)
> 10.0 g/d	1.94	(1.02 - 3.71)
	OR***	**95%CI**
Pre- and postmenopausal (n** = 420)		
Non-consumer	1 (ref.)	
0.1 - 5.0 g/d	1.48	(0.82 - 2.67)
5.0 - 10.0 g/d	1.64	(0.91 - 2.95)
> 10.0 g/d	1.47	(0.82 - 2.63)

Abbreviations: *n*, number of observations; *OR*, Odds Ratio; *95%CI*, 95% confidence interval; *g*, gram; *d*, day.

* Fully adjusted models were adjusted for physical activity, BMI, HRT, menopausal status, age, mother with history of breast cancer, school education, ever use of oral contraceptives, age at menarche, smoker at recruitment, number of live births, alcohol consumption.

**Numbers may not add up to 424 due to missing values.

*** Parsimonious models were adjusted for age and BMI at recruitment.

## Discussion

Adherence to Mediterranean diet was inversely associated with mammographic density. Our data show further that the consumption of multivitamin-multimineral supplements is associated with a reduction in mammographic density. High levels of alcohol consumption may be related to higher mammographic density.

### Mediterranean diet

In line with former research on diet and mammographic density, we found an inverse association between the adherence to Mediterranean diet score and mammographic density [11,19,20]. Our results did not change by additional adjustment for alcohol consumption and multivitamin-multimineral supplements.

Previous analyses of single food groups and mammographic density showed that components of the Mediterranean diet such as vegetables and olive oil could be inversely associated [20]. Consistent with our finding of an inverse association among postmenopausal women, prospective studies on breast cancer have shown that conformity to Mediterranean diet is associated with reduced breast cancer risk among postmenopausal women [17,21]. However, among postmenopausal women in Japan intakes of protein, total fat, and saturated fat were significantly positively associated with percentage of mammographic density [22], while carbohydrates were inversely associated. For components of the Mediterranean diet biological mechanisms have been described related to antioxidants, phytoestrogens and fatty acids [23]. In an observational study inverse associations between phytoestrogens and mammographic density have been reported [24].

In contrast to Tseng et al. (2008) we observed a statistically significant inverse association of Mediterranean diet score with mammographic density among non-smoking women.

### Multivitamin-multimineral supplements

Our findings of an inverse association between the current use of multivitamin-multimineral supplements and mammographic density in postmenopausal women are in contrast to results from a case–control study showing that multivitamin-multimineral supplement use is associated with higher breast density among premenopausal women [25]. Our results did not substantially change by additional adjustment for educational levels or other adjustment variables. Due to the antioxidant property of vitamins and minerals an inverse association seems to be biologically plausible [26].

Epidemiological studies on diet have shown inverse associations between vitamin D intake and mammographic density [27]. For the intake of vitamin C and E positive associations with mammographic density [19] and inverse associations for vitamin C intake have been reported [20].

Women who take multivitamin-multimineral supplement may have a healthier lifestyle than others. However, differences in the composition of the multivitamin-multimineral supplement pills and the inclusion of potential confounders could have influenced the associations. Data on use of vitamin and mineral supplements and its combination are scarce [25].

### Alcohol consumption

In our study, current alcohol intake of more than 10g/d was associated with high mammographic density in pre- and postmenopausal women. Results from prospective [20,28], case–control [29] and cross-sectional [30] studies have shown that increased alcohol intake is associated with mammographic more dense breast parenchyma. However, also no association between alcohol intake and mammographic density was found in a cross-sectional study [31]. Flom et al. investigated alcohol beverages and found that particularly beer and white wine consumption may increase mammographic density [28].

There is evidence linking alcohol by Insulin-like growth factor (IGF) and Insulin-like growth factor binding protein (IGFBP)-3 to mammographic density [32,33]. In addition, alcohol increases estrogen levels in blood [34] which were found to increase mammographic density [35].

### Strengths and limitations

One potential limitation of this study is that measurement error is inherent in dietary assessment. However, our data for alcohol were consistent with other data from Germany [16], and for dietary variables long-term reproducibility was fairly [36] indicating that dietary habits are stable over longer time intervals.

In the Mediterranean diet score in cases with no information on food items (e.g. for fish), 0 points were given. However, exclusion of individuals without information revealed similar results in the fully adjusted model without elimination of these women.

Since a cross-sectional study was performed, we cannot appraise the time sequence of the associations. The data on exposure variables were collected retrospectively, which may have introduced recall bias. However, it is likely to be non differential.

The assessment of the mammograms by experienced radiologists is one of the strengths of our study. In addition, we calculated the fully adjusted and the parsimonious models which revealed similar results but only statistical significance was reached in the parsimonious models.

The inter-rater reliability for the analyzed categories of ACR classification as indicator for mammographic density was good. ACR classification of mammographic density is widely applied and it has been shown that it is correlated with more refined measurement methods [8]. Information on reproductive and use of exogenous hormones has been collected by a standardized questionnaire. However, in our data these variables did not substantially affect the estimates.

## Conclusions

In summary, this study suggests that adherence to the Mediterranean diet and current use of multivitamin-multimineral supplements are associated with mammograhically less dense breast parenchyma as intermediary marker of breast cancer risk. Further studies are needed to investigate the value of dietary intervention in the prevention of breast cancer.

## Abbreviations

BI-RADS: The breast imaging reporting and data system; ACR-categories: Categories of the American College of Radiology; BMI: Body-mass-index; HRT: Hormone replacement therapy; SAS: Statistical analysis system; IGF: Insulin-like growth factor; IGFBP: Insulin-like growth factor binding protein.

## Competing interests

The authors declare that they have no competing interests.

## Authors’ contributions

GN, AB conception of the study; CB, BA data collection; OV, GN data analyses; OV wrote the first draft, which was refined by contributions of CB, BA, GN. All authors read and approved the final manuscript.

## Pre-publication history

The pre-publication history for this paper can be accessed here:

http://www.biomedcentral.com/1471-2458/13/203/prepub
